# Notes and News

**Published:** 1902-06-28

**Authors:** 


### Notes and News.

The advisory committee appointed in connection with the proposed King's Sanatorium of Consumption have purchased a site at Midhurst, Sussex.

It is very satisfactory to hear that Professor Virchow continues to make steady progress after his severe accident. He is now able to take fairly long walks, which shows great powers of recuperation for a man of his age.

It having been proposed by the Glamorgan County Council to pass a by-law prohibiting spitting in public rooms and vehicles, the Home Secretary has given his sanction to it on condition that "its operation is confined to public carriages, public waiting-rooms, public halls, and places of public entertainment. He thinks that the by-law cannot properly be made to apply to churches, chapels, schools, and shops."

It is with the greatest possible regret that we record the death of Dr. John Wychenford Washbourn, C.M.G., physician to Guy's Hospital and the London Fever Hospital, which took place at Tunbridge Wells on the 20th inst. To Guy's Hospital and its medical school his death will be an irreparable loss, and we are sure that the news of his decease will be a great shock to a very large number of men who came under his care at the Imperial Yeomanry Hospital at Deelfontein and Pretoria.

The British Medical Association has issued a tremendous programme for its Manchester meeting, containing perhaps nothing of striking novelty, but a long list of subjects of considerable interest. Dilatation of the Stomach leads the way on the medical side; and, on the surgical, the Treatment of Inoperable Cancer. The obstetrical section will deal with Cæsarean Section. Public medicine will discuss many topics, and among others the enormous subject of the Relation of Poverty to Disease. Among the readers of papers we note a considerable number of new names, and there seems much reason to hope that, notwithstanding all the little acerbities which at one time seemed likely to come unpleasantly to the fore, the meeting will be very successful.

The Worshipful Company of Fishmongers have made a donation of £21 to the funds of the After Care Association for Poor Persons Discharged Recovered from Asylums for the Insane.

Pending the erection of the new Medical Staff College in London for the officers of the Royal Army Medical Corps, some of the laboratories on the Victoria Embankment belonging to the Royal Colleges will be rented for purposes of post-commission study.

The object of the Cremation Bill, which now seems likely to become law, is to enable local authorities to erect crematoria without obtaining special Acts of Parliament for the purpose. The Bill also provides that wherever crematoria are established they shall be properly managed and equipped, and shall be inspected by the inspectors of the Home Department. The Home Office will be responsible for issuing rules as to their use.

An Act to establish a General Medical Council for Canada has been passed by the Canadian Legislature. The Act is permissive and cannot take effect until the consent of the several provincial legislatures has been obtained, and this may take time. When, however, it comes into operation, it will do more than merely set up a general standard of medical education in Canada, for as a secondary result it will, it is hoped, make it possible to establish reciprocity with England, which is impossible so long as there is no medical qualification in Canada which is good for the whole Dominion.

Although it is improbable that the Bill to amend the Cruelty to Animals Act, 1876, will make any further progress this Session, it is worth noting that the provisions which it contains are such that if passed it would not only put a stop to nearly all useful physiological inquiry, but would place great difficulties in the way of the investigation of pathological problems. Doubtless this is what is aimed at by those responsible for the measure. It must, however, be borne in mind that pleasant as it may be to certain people to impose these obstructions in the path of the scientific investigation of disease, disease will always be present in our midst, and that if we will not investigate we must suffer.

The concentration of a large number of troops in the neighbourhood of London on the occasion of the Coronation has necessitated the organisation of a series of hospitals for their use. At the Alexandra Palace, where two or three thousand Colonial and native troops have been assembled, three temporary hospitals, providing altogether 52 beds, have been established, and arrangements have been made for the transport of the more serious cases by invalid railway-carriage to various hospitals in London. At Hampton Court, where the Indian contingent is located, there is a field hospital of 50 beds; and there is a small detention hospital at Fulham. Another field hospital of 50 beds is established at Hounslow Heath, arrangements being made for the reception of serious cases at the station hospital there. Sick European officers are treated at St. Thomas's Home, while infectious cases are removed to the civil isolation hospitals.

